# Optimising antiretroviral therapy through a proactive treatment algorithm: a cost-effective strategy in Dutch healthcare for people with HIV

**DOI:** 10.1093/jac/dkaf117

**Published:** 2025-04-12

**Authors:** P Oosterhof, M van Luin, K Grintjes, N van der Meche, R van Crevel, K Brinkman, D M Burger

**Affiliations:** Department of Clinical Pharmacy, OLVG Hospital, Amsterdam, The Netherlands; Department of Pharmacy, Radboudumc Research Institute for Medical Innovation (RIMI), Radboud University Medical Center, Nijmegen, The Netherlands; Department of Clinical Pharmacy, Meander Medical Center, Amersfoort, The Netherlands; Department of Internal Medicine, Radboud Center for Infectious Diseases, Radboud University Medical Center, Nijmegen, The Netherlands; Division of Infectious Diseases, Department of Internal Medicine, OLVG, Amsterdam, The Netherlands; Department of Internal Medicine, Radboud Center for Infectious Diseases, Radboud University Medical Center, Nijmegen, The Netherlands; Division of Infectious Diseases, Department of Internal Medicine, OLVG, Amsterdam, The Netherlands; Department of Pharmacy, Radboudumc Research Institute for Medical Innovation (RIMI), Radboud University Medical Center, Nijmegen, The Netherlands

## Abstract

**Background:**

A wide range of effective antiretroviral therapy (ART) regimens with favourable side effects are available. More than 70% of HIV care costs in the Netherlands are attributed to ART. We developed an ART algorithm to proactively switch virologically suppressed individuals to a more cost-effective HIV treatment.

**Methods:**

This prospective study implemented our ART algorithm in two large Dutch HIV clinics, where a pharmacist screened ART regimens for 1 year. Individuals were considered suitable for a switch if their current ART exceeded €600 per month, considering renal function and/or tubular toxicity, hepatitis B status, and resistance history. If eligible, advice with a switch proposal was recorded in the patient file. The objective was to investigate the acceptance of the proposal and the effect of proactive switching on the total costs of ARTs.

**Findings:**

Of 1596 people living with HIV, 840 (52.6%) were eligible. Prescribers accepted 81.1% of the switch proposals, and 84.9% of eligible individuals agreed to the proposed switch. Ultimately, 558 individuals accepted the new ART regimen proposal, with doravirine/tenofovir disoproxil fumarate/lamuvidine (DOR/TDF/3TC) being the most prescribed (61.6%). The switch led to significant cost savings, reducing annual ART expenditure from €10 923 to €8580 per eligible individual, totalling almost €2 million (−21.4%) in savings annually.

**Interpretation:**

Our ART algorithm demonstrated high acceptance by prescribers and people with HIV, leading to substantial cost savings. The algorithm can be easily implemented in other HIV clinics to offer even more significant cost savings to Dutch healthcare payers.

## Introduction

The management of HIV as a chronic condition has been transformed by antiretroviral therapy (ART), leading to normalising life expectancies for people living with HIV.^1^ In the Netherlands, the population of people living with HIV is expanding and becoming older, placing a significant financial burden on healthcare resources.^2,3^ As of 2022, an estimated 24 400 individuals are living with HIV in the Netherlands,^2^ underscoring the need for strategies that optimise ART costs without compromising the quality of treatment.

The financial implications of HIV and AIDS healthcare were €201.7 million in 2019, of which 70% (141 million) was attributed to ART.^4^ The cost burden is high owing to the diverse pricing of ART medications, many of which have comparable short-term efficacy and tolerability, though long-term safety profiles may differ.^5^ A trend towards the adoption of single-tablet regimens (STRs) and integrase strand transfer inhibitors (INSTIs) has been noted for their clinical benefits, although they come at higher costs. By 2022, STRs represented 60.1% of ART usage, increasing the financial burden on the healthcare system.^2^ Traditionally, treatment changes have been reactive, focusing on clinical needs rather than costs, highlighting the urgency for innovative cost-saving approaches.^6^

Cost-saving prescribing practices have become increasingly crucial for reducing the overall expenses associated with HIV care. Since 2017, the introduction of widely used HIV generic drugs in high-income countries has offered the opportunity to reduce medication costs.^7,8^ These generics facilitated two primary strategies: the direct substitution of brand-name medications,^8,9^ and the splitting of STRs into two or more pills, using inexpensive generic backbones.^10–14^ However, the continued increase in the use of STRs, coupled with the challenge that some STRs cannot be deconstructed using cheaper generic combinations, has limited the impact of these cost-saving methods. This development underscores the need for innovative, cost-reducing strategies.

The current ART landscape in the Netherlands features a wide selection of effective STRs at competitive prices. One way to maximize the use of cost-effective STRs is to proactively switch virologically suppressed individuals to a more cost-effective ART regimen. In the Netherlands, all residents are required to have health insurance, and ART is fully reimbursed under the national healthcare system, with patients having the freedom to obtain their medication from a pharmacy of their choice.^15^ With the development of a treatment algorithm using cost-effective STRs, significant savings can be achieved while maintaining or even improving quality of care.

We initiated a multicenter study in the Netherlands in which we introduced a proactive treatment algorithm aimed at optimising ART costs within the Dutch healthcare context. The algorithm leverages the availability of well-priced but effective STRs and suggests therapeutic changes that maintain clinical integrity while reducing the financial burden.

## Methods

### Study design

We conducted a multicenter, prospective cohort study across two hospitals in the Netherlands: the Radboud University Medical Center (Radboudumc) in Nijmegen and the OLVG hospital in Amsterdam. These centers provide care for over 4500 people with HIV, representing approximately 20% of the total HIV population in the Netherlands.^2^ A treatment algorithm was designed with the goal of reducing healthcare costs by switching treatment-experienced people with HIV to more cost-effective STRs and was implemented across the two HIV treatment centers. This study aimed to evaluate a proactive treatment algorithm rather than reassess the clinical effectiveness of ART regimens, as all prescribed ART regimens have been extensively studied and demonstrated sustained virological efficacy in real-world settings. This study was conducted between January 2020 and January 2021.

The primary objective was to assess both prescriber and patient acceptance of the proposed proactive treatment algorithm for switching treatment-experienced individuals to cost-effective ART regimens. The secondary objectives included the evaluation of therapy persistence within 12 months post-switch, in addition to calculating the cost savings achieved by switching to less expensive ART regimens.

### Patient selection

Potential participants were screened and assessed for eligibility through a comprehensive review of their medical history and current health status by a multidisciplinary treatment team. Individuals living with HIV, aged 18 years or older, and on ART at a monthly cost of at least €600 were considered eligible for inclusion. A threshold of €600 per month was chosen because the cheapest STR at the initiation of this study was doravirine/tenofovir disoproxil fumarate/lamivudine (DOR/TDF/3TC) at a cost of approximately €500 per month. A monthly difference of €100 was considered acceptable as the minimum for the efforts to be taken.

The eligibility criteria further specified that patients must have maintained a virologically suppressed status, defined as having an undetectable viral load for at least 12 months prior to being considered for the study. The patients had to use their current ART treatment for more than 1 year. Additional considerations included resistance profiles, presence of comorbidities—particularly those affecting renal function and/or indicative of current or past tubular toxicity—and co-infection with hepatitis B. These considerations align with existing treatment guidelines and routine clinical decision-making in HIV care.

Individuals with a history of treatment failure, known allergies to components of the proposed medication options, or significant issues with medication adherence were excluded. These individuals were deemed to be ineligible for the switch proposal.

### Treatment algorithm

**Figure 1.**
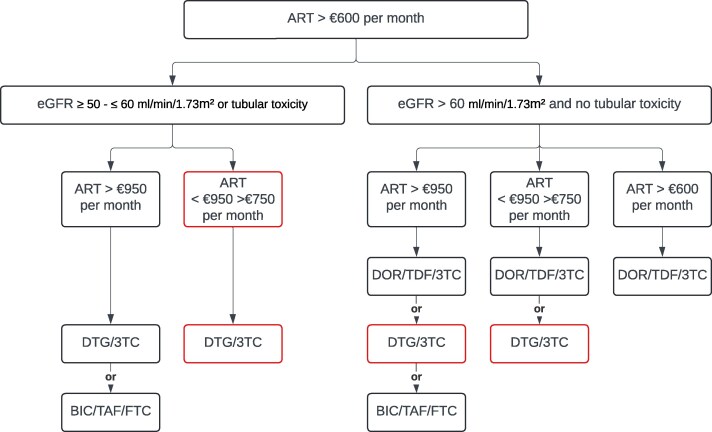
Antiretroviral treatment algorithm. 3TC, lamivudine; BIC, bictegravir; DOR, doravirine; DTG, dolutegravir; eGFR, estimated Glomerular Filtration Rate; FTC, emtricitabine; TAF, tenofovir alafenamide; TDF, tenofovir disoproxil fumarate. Source: Medicines Evaluation Board http://english.cbg-meb.nl/ and European Medicines Agency, http://www.ema.europa.eu/. The option in red expires as soon as a patient has a hepatitis B co-infection, and who must therefore be treated with the presence of tenofovir disoproxil fumarate or tenofovir alafenamide fumarate in accordance with the treatment guidelines.

The treatment algorithm was specifically designed to identify individuals eligible for switching to a more cost-effective STR (Figure 1). The selected STRs were bictegravir/tenofovir alafenamide fumarate/emtricitabine (BIC/TAF/FTC; Biktarvy^®^), DOR/TDF/3TC (Delstrigo^®^), and dolutegravir/lamivudine (DTG/3TC; Dovato^®^). These particular STRs were chosen based on the criteria outlined in Table 1, prioritising regimens that required no food restrictions for intake, did not have a booster (cobicistat) to avoid drug-drug interactions, and supporting oral-to-oral switches, while also ensuring competitive pricing. This rationale narrowed the selection to the mentioned STRs. The treatment algorithm included additional cost thresholds (€950 and €750 per month) to guide regimen selection based on available ART pricing. These thresholds were established considering the costs of BIC/TAF/FTC (€860), DTG/3TC (€673), and DOR/TDF/3TC (€486), ensuring that each switch met the predefined requirement of achieving at least €100 in monthly cost savings.

**Table 1.** STRs for HIV treatment in the Netherlands as of 2020, with price (€) and approval dates

STR (brand-name)	Authorisation date	Price (€)	Limiting factor
DOR/TDF/3TC (Delstrigo^®^)	22 November 2018	486.60	
DTG/3TC (Dovato^®^)	03 July 2019	673.36	
RPV/TAF/FTC (Odefsey^®^)	21 June 2016	686.08	Food restriction
RPV/TDF/FTC (Eviplera^®^)	28 November 2011	718.27	Food restriction
DTG/RPV (Juluca^®^)	21 May 2018	799.00	Food restriction
DRV/c/TAF/FTC (Symtuza^®^)	21 September 2017	846.28	Booster
BIC/TAF/FTC (BIktarv^®^)	25 June 2018	860.00	
DTG/ABC/3TC (Triumeq^®^)	01 September 2014	886.18	Price
EVG/c/TAF/FTC (Genvoya^®^)	19 November 2015	893.12	Price/booster
EVG/c/TDF/FTC (Stribild^®^)	24 May 2013	969.50	Price/booster

Source: Medicines Evaluation Board http://english.cbg-meb.nl/ and European Medicines Agency, http://www.ema.europa.eu/.

3TC, lamivudine; ABC, abacavir; BIC, bictegravir; c, cobicistat; DOR, doravirine; DRV, darunavir; DTG, dolutegravir; EVG, elvitegravir; FTC, emtricitabine; TAF, tenofovir alafenamide; TDF, tenofovir disoproxil fumarate; RPV, rilpivirine.

A pharmacist, who was a member of the multidisciplinary treatment team, applied the algorithm to make an initial recommendation, which was noted as a predefined note in the electronic patient file. Physicians or nurse specialists can access these recommendations before or during consultations, independently evaluating whether to present the advised switch to the patient. If no response to the initial recommendation could be found in the electronic file after 6 months, the pharmacist entered a repeated recommendation into the patient file as a reminder.

Patients were not forced to make a switch; they retained the freedom to continue their existing ART regimens. This decision was supported by transparent communication of the cost differences between the regimens, highlighting that while switching would not result in direct financial benefits to the patients themselves, it could contribute to healthcare cost savings on a societal level. Any decisions against switching were documented, and people were assured of the option to revert to their previous regimen, if desired.

### Data collection

The data collected included HIV diagnosis date, ART initiation date, switch date to a new regimen, viral load, CD4 count, recorded adverse events, and co-medication. Virological response was assessed through routine viral load monitoring, with virological suppression defined as an HIV RNA level <50 copies/mL. Viral load data were collected at baseline and during follow-up as part of standard care. Patient persistence—defined as the continuation of therapy from initiation to discontinuation—was monitored for 12 months post-switch through the hospital's electronic patient file system, enabling the tracking of treatment continuity. Prices for various antiretroviral therapies were obtained from the Dutch National Website for Drug Prices (www.medicijnkosten.nl). To ensure accurate financial analyses, costs were calculated based on prices for the corresponding years of the study period.

### Data analysis and statistics

Exploratory data analysis methods were employed to evaluate changes over time, including acceptance rates of both prescribers and patients, persistence with the new regimen, and cost savings. This study used descriptive analyses to focus on the impact of ART switching on treatment persistence and ART costs. Persistence was assessed using the electronic patient files of the hospital, supplemented by clinical documentation.

The cost analysis focused on ART costs, which account for approximately 70% of the total HIV treatment expenditures.^4^ To ensure the precision and relevance of our financial assessment, pharmacy dispensing fees and medication waste were excluded. This decision was made considering their minimal impact on the overall healthcare budget, allowing us to concentrate on the main factors that influence healthcare costs.

### Ethics

This study was classified as non-WMO (Medical Research Involving Human Subjects Act) applicable, individual patient consent was not required. The intervention was considered part of standard HIV care within these hospitals. Ethical approval was obtained from the Medical Ethical Committee of Radboudumc in Nijmegen, Netherlands (approval number 2020-6299).

## Results

### Overview of study enrolment and participant characteristics

**Figure 2.**
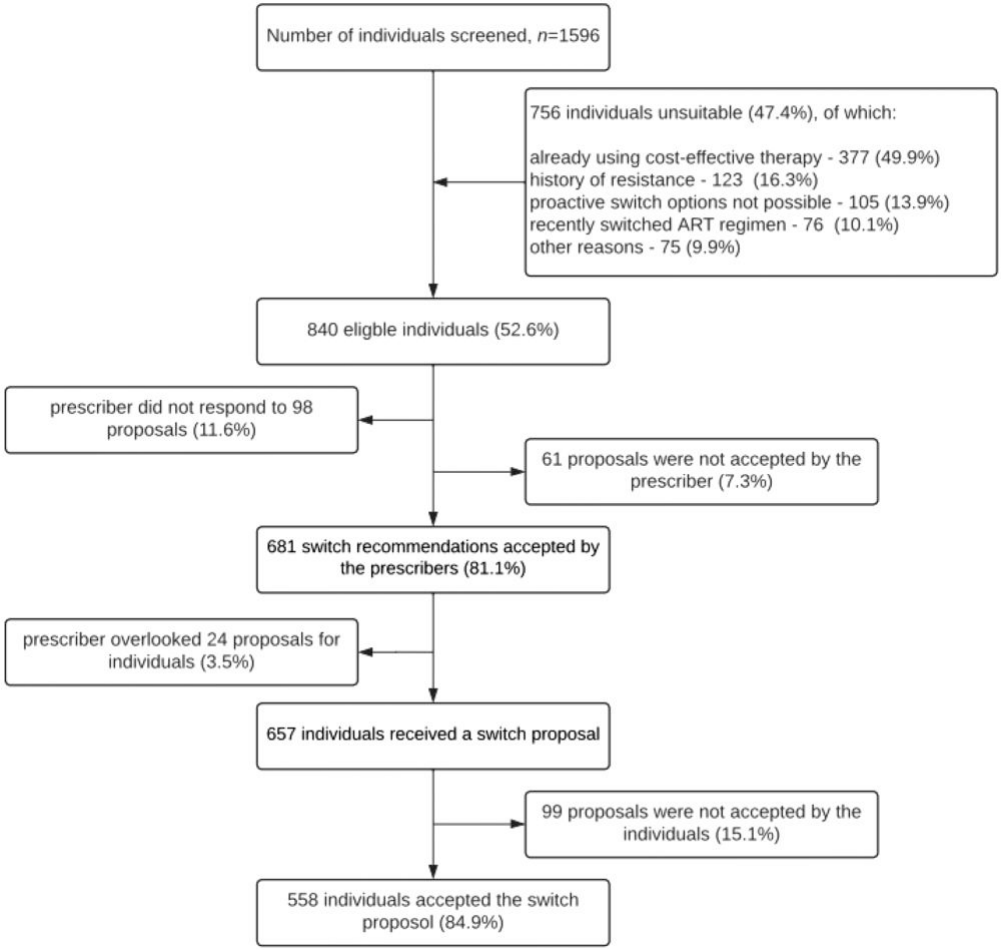
Selection of treatment-experienced people with HIV and acceptation of the switch proposals.

In this prospective study conducted at two Dutch HIV treatment centers, we screened 1596 people with HIV for a proactive switch to more cost-effective ART regimens (Figure 2). Of these, 756 individuals (47.4%) were not eligible for various reasons: 377 (49.9%) were already using a cost-effective therapy; 123 (16.3%) had a history of resistance, specifically to one or more components of the proposed switch regimens; 105 (13.9%) were not suitable for the chosen regimen because of reasons such as the desire to become pregnant or a combination of impaired renal function and the cost implications of a regimen change; 76 (10.1%) had recently switched ART regimens; and 75 (9.9%) were excluded for other reasons such as low-level viremia, tube feeding, serious illness, and burdensome comorbidities.

The remaining 840 individuals (52.6% of the initially screened population) were considered eligible for inclusion in the study. A substantial majority of the individuals were male, constituting 88.5% (*n* = 743) of this group (Table 2). The median age was 52 years. These individuals had been diagnosed with HIV for a median of 12 years and had been on ART for a median of 10.5 years. The median duration since the initiation of the current ART regimen was 4 years. Regarding co-medication, the median number of concurrent medications taken by individuals was two. The study population came from two centres, with 490 (58.3%) enrolled from OLVG and 350 (41.7%) from RadboudUMC. For combinations of ART, 579 (68.9%) individuals were on STRs, with the most common being dolutegravir/abacavir/lamivudine (DTG/ABC/3TC; Triumeq^®^) (22.9%), elvitegravir/cobicistat/tenofovir alafenamide fumarate/emtricitabine (EVG/c/TAF/FTC; Genvoya^®^) (17.5%), and BIC/TAF/FTC (14.5%). A smaller proportion of individuals used multi-tablet regimens (MTRs), with DTG + TDF/FTC and DTG + TAF/FTC being favoured, representing 10.5% and 7.5%, respectively.

**Table 2.** Baseline characteristics of the eligible individuals

Parameter	Total (*n* = 840)
Sex assigned at birth, *n (%)*	
Male	743 (88.5)
Female	97 (11.5)
Age, year	
Median (IQR)	52 (42–60)
Time since HIV diagnosis, years	
Median (IQR)	12 (7–18)
Time since first ART, years	
Median (IQR)	10.5 (6–16)
Time since current ART, years	
Median (IQR)	4 (2–5)
Co-medication	
Median (IQR)	2 (1–5)
Centre, *n* (%)	
OLVG	490 (58.3)
RadboudUMC	350 (41.7)
Number of ART tablets	
Median (IQR)	1 (1–2)
Combinations of ART, *n* (%)	
*Single-tablet regimens*	
DTG/ABC/3TC	192 (22.9)
EVG/c/TAF/FTC	147 (17.5)
BIC/TAF/FTC	122 (14.5)
RPV/TAF/FTC	52 (6.2)
STR with a TAF/FTC backbone	34 (4.0)
STR with a TDF/FTC backbone	25 (3.0)
Other STRs	7 (0.8)
*Multi-tablet regimens*	
DTG + TDF/FTC	88 (10.5)
DTG + TAF/FTC	63 (7.5)
MTR with a TAF/FTC backbone	61 (7.3)
MTR with a TDF/FTC backbone	28 (3.3)
Other MTRs	21 (2.5)

3TC, lamivudine; ABC, abacavir; ART, antiretroviral therapy; BIC, bictegravir; c, cobicistat; DOR, doravirine; DTG, dolutegravir; EVG, elvitegravir; FTC, emtricitabine; IQR, interquartile range; MTR, multi-tablet regimen; RPV, rilpivirine; STR, single-tablet regimen; TAF, tenofovir alafenamide; TDF, tenofovir disoproxil fumarate.

### Acceptance of a proactive treatment algorithm

Among the 840 individuals deemed eligible for the study, initial engagement by the prescribers resulted in 98 proposals (11.6%) that did not receive any response in the electronic patient file. In total, 61 proposals (7.3% of all eligible individuals) were not accepted by the prescribers, often due to additional information they had about the patient, such as fluctuating outpatient visits, complicating comorbidities, or acute changes in clinical parameters, such as kidney function or general condition. Ultimately, 681 switch recommendations were accepted by the prescribers, resulting in a prescriber acceptance rate of 81.1% for proactive ART switching. However, within this group, 24 proposals were inadvertently not communicated with their patients, accounting for 3.5% of the cohort. This oversight was often due to many topics that had to be discussed during outpatient visits. As a result, 657 individuals ultimately received a switch proposal that was reviewed and was considered actionable. Of these, 99 proposals (15.1%) were declined by the individuals themselves for various personal reasons, such as satisfaction with their current ART and fear of potential side effects. Consequently, 558 individuals accepted the switch proposal and switched to the new ART regimen, leading to an acceptance rate of 84.9% among people living with HIV.

### Algorithm outcomes

Table 3 outlines the specific ART switch outcomes among the 558 individuals who accepted a proactive ART switch proposal. DOR/TDF/3TC emerged as the most prescribed option and was selected in 343 individuals (61.5%). This group was generally younger, with a median age of 48 years and a median duration since HIV diagnosis of 10 years. They had fewer co-medications and predominantly transitioned from STRs, notably from EVG/c/TAF/FTC (19.2%) and DTG/ABC/3TC (16.3%). DTG/3TC was proposed for 185 individuals. This group had an older median age of 55 years and a longer median duration since HIV diagnosis of 13 years. A large number of these individuals (51.9%) had previously been on DTG/ABC/3TC. BIC/TAF/FTC was prescribed to 30 individuals, representing the oldest subgroup with a median age of 57 years and the longest median duration since HIV diagnosis at 14 years. This regimen was predominantly selected in individuals with a higher number of co-medications, with the majority transitioning from MTRs, specifically DTG + TAF/FTC (53.3%). Among those who switched to the new regimens, no patient failed treatment based on viral load and CD4 count data in this observed analysis approach, demonstrating the clinical effectiveness of the proactive switch. Notably, three individuals did not proceed with starting the new STR for specific reasons: one had a TB infection, another experienced a viral blip prior to the switch, and the third went on a long journey abroad. Additionally, two patients with HBV co-infection who received DTG/3TC were on separate hepatitis B treatments to ensure adequate viral suppression.

**Table 3.** characteristics of the switch population per ART treatment choice

Parameter	DOR/TDF/3TC (*n* = 343)	DTG/3TC (*n* = 185)	BIC/TAF/FTC (*n* = 30)
Sex assigned at birth, *n (%)*			
Male	298 (86.9)	170 (91.9)	28 (93.3)
Female	45 (13.1)	15 (8.1)	2 (6.7)
Age, year			
Median (IQR)	48 (39–48)	55 (47–55)	57 (51–57)
Time since HIV diagnosis, years			
Median (IQR)	10 (6–15.5)	13 (8–17)	14 (9–23)
Time since first ART, years			
Median (IQR)	9 (5–13)	11 (6–16)	13 (8.25–17)
Time since current ART, years			
Median (IQR)	4 (2–5)	4 (3–5)	3.5 (3–5)
Co-medication			
Median (IQR)	2 (1–4.5)	2 (1–5)	4 (1–7)
Center, *n* (%)			
OLVG	201 (58.6)	102 (55.1)	6 (20.0)
RadboudUMC	142 (41.4)	83 (44.9)	24 (80.0)
Number of ART tablets			
Median (IQR)	1 (1–2)	1 (1–1)	2 (2–3)
HBV co-infection	31 (9.0)	2 (1.1)	8 (26.7)
eGFR >60	335 (97.7)	136 (73.5)	16 (53.3)
eGFR <60	8 (2.3)	49 (26.5)	14 (46.7)
Tubular toxicity	2 (0.6)	24 (12.9)	13 (43.3)
Combinations of ART, *n* (%)			
*Single-tablet regimens*			
DTG/ABC/3TC	56 (16.3)	96 (51.9)	—
EVG/c/TAF/FTC	66 (19.2)	29 (15.7)	3 (10.0)
BIC/TAF/FTC	30 (8.7)	21 (11.4)	—
RPV/TAF/FTC	35 (10.2)	2 (1.1)	—
STR with a TAF/FTC backbone	19 (5.5)	4 (2.2)	1 (3.3)
STR with a TDF/FTC backbone	19 (5.5)	—	—
Other STRs	2 (0.6)	1 (0.1)	—
*Multi-tablet regimens*			
DTG + TDF/FTC	57 (16.6)	10 (5.4)	—
DTG + TAF/FTC	13 (3.8)	15 (8.1)	16 (53.3)
MTR with a TAF/FTC backbone	14 (4.1)	5 (2.7)	10 (33.3)
MTR with a TDF/FTC backbone	20 (5.8)	—	—
Other MTRs	12 (3.5)	2 (0.2)	—
*Never started the proposed ART*	1 (0.3)	2 (1.1)	
*Persistence, who discontinued*			
Stopped within 12 months, *n* (%)	35 (10.2)	9 (4.9)	8 (26.7)
Time until discontinuation, *n* (median, IQR)	3 (2–5)	1 (1–5.5)	3 (2.75–3.25)

3TC, lamivudine; ABC, abacavir; ART, antiretroviral therapy; BIC, bictegravir; c, cobicistat; DOR, doravirine; DTG, dolutegravir; EVG, elvitegravir; FTC, emtricitabine; IQR, interquartile range; MTR, multi-tablet regimen; STR, single-tablet regimen; TAF, tenofovir alafenamide; TDF, tenofovir disoproxil fumarate; RPV, rilpivirine.

Persistence rates among the three selected ART regimens varied, with overall rates ranging from 73.3% to 95.1%. Among those who started DOR/TDF/3TC, 35 individuals (10.2%) discontinued the regimen within 12 months, with a median time to discontinuation of 3 months. In the DTG/3TC group, nine individuals (4.9%) stopped treatment within 12 months, with a median time to discontinuation of one month. The BIC/TAF/FTC group showed the highest discontinuation rate, with eight participants (26.7%) discontinuing within 12 months and a median time to discontinuation of 3 months.

### Expenditure on ART

**Figure 3.**
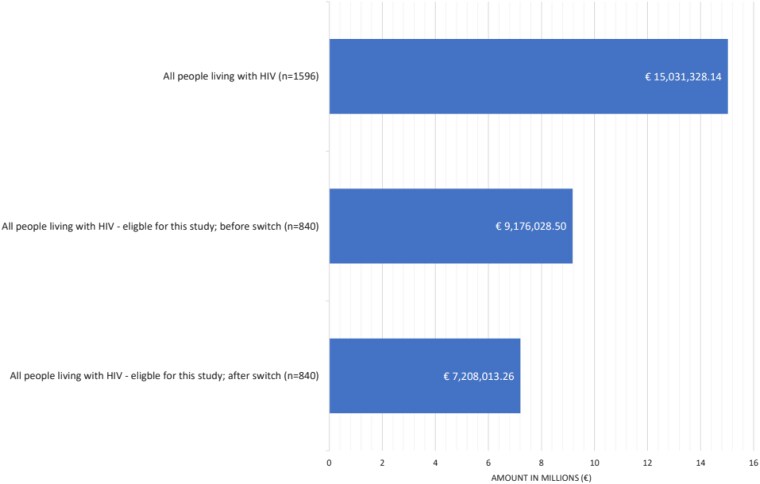
Expenditure on ART per year for the different groups.

Figure 3 shows the financial implications of the proactive ART switch by examining annual expenditures across different groups. Across the entire cohort of 1596 individuals assessed in the study, the total annual expenditure on ART (prior to a potential switch) was €15.03 million, averaging €9418 per individual per year. For the 840 individuals eligible for the switch, the financial analysis before the switch showed an average yearly expenditure of €9.17 million on ART, or €10 923 per individual per year. After implementing the proactive switch to more cost-effective regimens, this expenditure decreased to €7.20 million annually, equal to €8580 per individual per year (−21.4%).

## Discussion

Our multicenter study evaluated a proactive treatment algorithm to optimise ART towards more cost-effective regimens in the Dutch healthcare system. Of the 840 eligible people living with HIV, 81.1% of the switch proposals were accepted by prescribers, and 84.9% of the individuals proposed a switch agreed; DOR/TDF/3TC was the most prescribed switch regimen. Implementing this proactive treatment algorithm substantially reduced the total ART expenditure, resulting in annual savings of almost 2.0 million euros across the entire cohort of 840 individuals (−21.4%).

Our findings on proactively switching virologically suppressed people with HIV to more cost-effective regimens without compromising therapeutic outcomes offer a pragmatic approach to the challenge of managing increasing costs.^16^ Furthermore, our results on acceptance and cost savings are a new contribution to the existing literature on HIV care management, because there are no comparable studies in this field.

The shift towards STRs and INSTIs represents a move towards simplification and efficacy in ART, although with cost implications.^17,18^ Previous research has primarily focused on the introduction of generic medications and the direct substitution of brand-name drugs to reduce costs. International studies have shown that it is possible to reduce costs by asking people with HIV to switch their STR to a MTR consisting of the same components but with a generic backbone.^11,13,19^ These studies, and our own multicenter study, showed that in general, more than 50% of individuals are open to this switch and that this can save around 18% of the costs of ART.^20^ Other studies have focused on cost savings from switching patients from STR consisting of three or more components to dual therapy, such as DTG/3TC.^21,22^ Depending on the original STR regimen, switching to DTG/3TC resulted in cost savings ranging between 16% and 26%. Our approach differs by systematically applying a proactive treatment algorithm across a patient cohort, emphasising the optimisation of existing STRs for cost-efficiency.

Switching individuals from DTG/ABC/3TC (Triumeq^®^) to DTG/3TC (Dovato^®^) is currently straightforward, although alternatives such as DOR/TDF/FTC (Delstrigo^®^) or a MTR using branded DTG with a generic ABC/3TC backbone also exist. By integrating our proactive treatment algorithm and generic substitution, it is possible to achieve maximum cost-efficiency in ART prescribing. For instance, individuals on DTG/ABC/3TC could continue with a STR, such as DTG/3TC or DOR/TDF/3TC, or switch to a MTR, depending on their clinical needs and financial considerations. The financial benefits of combining these strategies could be substantial: switching to a MTR could lead to a 22% cost reduction, while switching from STRs to DTG/3TC might yield a 14% reduction, and switching to DOR/TDF/3TC, a 38% reduction. These variations underscore the importance of educating both patients and prescribers about the economic impact of their treatment choices. Proactive patient involvement in decision-making enhances awareness of therapeutic options and costs, improving adherence by aligning treatments with individual preferences and financial capabilities.^23^ Prescribers must also be well informed about the cost implications of different ART strategies, and compliance with updated evidence-based guidelines is crucial for aligning physician practices with cost-effective treatment strategies.^23^

The demonstrated annual cost-saving of almost €2.0 million in our patient population underscores the potential of targeted interventions in ART regimen to improve financial pressures on healthcare systems. By extrapolating these results to the broader context of the Netherlands, such strategies could potentially save up to €12.5 million annually while maintaining clinical efficacy. This is particularly relevant in a high-income country such as the Netherlands, where healthcare spending is aimed at ensuring both efficacy and cost.^24^ The high acceptance rate of the switch proposals indicates a strong patient trust in healthcare provider recommendations, reinforcing the feasibility of implementing such cost-optimisation strategies on a broader scale.^25^ The high acceptance rates observed in our study contrast with some previous findings where patients refuse to accept changes in therapy due to concerns over efficacy or convenience, which posed significant barriers.^26^ This discrepancy could be attributed to the comprehensive patient education and involvement in decision-making processes in our study, highlighting the importance of transparency and trust in healthcare provider-patient relationships.^27^

We concentrated on analyzing medication costs, which constitute approximately 70% of the total HIV care expenditure in the Netherlands.^4^ To maintain a focused analysis, we intentionally excluded costs such as pharmacy dispensing fees, additional tests (e.g. viral load), and personnel costs associated with the switch (e.g. prescribers, pharmacists, and nursing specialists). This decision was based on the assessment that these costs, while important, were minor compared with medication expenses. The study protocol was designed to ensure that individuals transitioned to new therapies only after their existing ART was completed. This measure was taken to prevent wastage and underscored that the switch was driven by cost-efficiency considerations rather than immediate clinical need.

The implications of our study extend beyond the Dutch healthcare system, offering a model for other countries that struggle with similar challenges in HIV care management. By demonstrating the feasibility and benefits of a proactive treatment algorithm, our research encourages a more dynamic approach to ART regimen selection that balances clinical needs with economic considerations. Healthcare systems worldwide can adapt this model, considering local drug availability, costs, and patient demographics, to enhance both the affordability and accessibility of HIV treatment. Furthermore, our study underscores the role of multidisciplinary collaboration in implementing such algorithms, involving pharmacists, clinicians, and patients. This collaborative approach not only facilitates the identification of suitable candidates for regimen switches, but also ensures that the changes are acceptable and beneficial from a patient perspective.

Although our study offers valuable insights, it is not without limitations. Our study could not determine why some clinicians and individuals did not accept or receive a proposal. Understanding the difficulties underlying the decision-making processes of prescribers and people with HIV requires further qualitative research. Additionally, the selection criteria may have introduced biases that affect generalizability. A predefined cost threshold of €600 for regimen inclusion may have influenced regimen selection, potentially favouring specific cost-effective STRs. While this approach was necessary to ensure cost savings, it could introduce selection bias and limit the generalizability of our findings to settings with different ART pricing structures. Furthermore, we focused on medication persistence rather than adherence, as refill data was not systematically available due to medication dispensing across multiple pharmacies. This limits the ability to assess whether persistence directly translates to adherence and real-world drug utilisation patterns. The generalizability of our findings to other healthcare settings, especially in other high-income countries, may be limited by differences in healthcare infrastructure, insurance, and patient populations. Furthermore, the long-term effects of the proposed treatment switches on patient outcomes, beyond cost savings, require further investigation. The focus of our study on virologically suppressed patients also raises questions about the applicability of the algorithm to individuals with more complex treatment histories or those not yet virally suppressed. Additionally, the reliance on data from the two treatment centres may not fully capture the broader spectrum of experiences and outcomes across the entire Dutch healthcare system. Future research could focus on exploring the cost implications of such a proactive approach in the Netherlands, as well as evaluating the impact of direct pharmacist-patient interactions in ART switch programs to determine whether pharmacist-led consultations improve patient engagement, therapy adherence, and long-term persistence.

### Conclusion

Our study provides a new perspective in the ongoing debate on ART cost optimisation by demonstrating the potential of a proactive, algorithm-based strategy to achieve substantial savings without compromising the quality of care. As healthcare systems continue to navigate the challenges of providing high-quality HIV care within financial limits, our findings underscores the importance of innovative approaches that prioritize economic efficiency.

## Data Availability

The data that support the findings of this study are available on request from the corresponding author. The data are not publicly available due to privacy or ethical restrictions.
